# Supplementary material for: Efficiency of antioxidant Avenanthramide-C on high-dose methotrexate-induced ototoxicity in mice
Source: PLoS One. 2022 Mar 30;17(3):e0266108. doi: 10.1371/journal.pone.0266108 (PMC8967015; doi:10.1371/journal.pone.0266108)
Supplement: Attachment — Submitted filename: CNUNI_5977_Response+to+Reviewers.v17.doc [file pone.0266108.s010.doc]

**Efficiency of Antioxidant Avenanthramide-C on High-Dose Methotrexate-Induced Ototoxicity in Mice**

**PONE-D-21-29604**

Thank you for giving us the opportunity to submit a revised draft of our manuscript titled “Efficiency of Antioxidant Avenanthramide-C on High-Dose Methotrexate-Induced Ototoxicity in Mice” to *PLOS ONE*. We truly appreciate the time and effort of the reviewers for providing their valuable feedbacks on our manuscript. We have been able to incorporate relevant changes to reflect most of the suggestions provided by the reviewers. We have highlighted all the changes in the revised manuscript. Here is a point-by-point response to the reviewers’ comments and concerns.

**Reviewer #1**

**Comment:**

**Q1-1. Synaptic ribbons: the authors used the term “synapses” in the manuscript, which is misleading since only presynaptic ribbons (CtBP2) were assessed (as described in the methods section). Synapses indicate both presynaptic ribbons (CtBP2) conjugated with post synaptic terminals (GluA2). The description of the number of presynaptic ribbons in the results section (lines 339–343) is also confusing. In general, the ribbon number should be calculated per inner hair cell, while Fig. 4B, C, and D are labeled as “Synapses/20 µm”. I suggest that the authors review pertinent articles, such as Kohrman et al., 2020, Kujawa and Liberman 2009 and recalculate the presynaptic ribbon number.**

**Response:**

We appreciate the reviewer's insightful feedback and ideas for improving our manuscript. As a result, we have ensured a high level of clarity in the updated manuscript addressing the synaptic ribbons counts and representation (page 7, lines 196–205). In our study, Figs. 4B, C, and D were labeled as "synaptic ribbons per IHC". In the corrected text, we used ‘ribbon synapses’ rather than ‘cochlear synapses’ (page 6, line 177) and corrected ‘tip’ to ‘apex’ (page 7, line 184). In addition, to avoid confusion and improve clarity, we used the terms synaptic ribbons to describe (CtBP2 punctates) throughout the paragraph entitled “*AVN-C treatment inhibits MTX-induced synaptic ribbons damage*” (page 12, line 339-353), as well as (page 17, line 486; page 18, lines 522,523,542; page 19, line 544; page 20, line 591), and at (page 25, lines 778, 779, 781, 782, 786,788). In the updated version, we replaced the statement "*MTX causes loss of synapses from the cochlear IHC bands*" (page 12, line 332-333) with " *MTX causes loss of synaptic ribbons of the cochlea IHC bands* " (page 12, line 344-345).

*.*

1. ***Counting of presynaptic ribbons of inner hair cells***

*Cochlear turn lengths were determined for each study group. A high-resolution confocal microscope (LSM 800 laser scanning microscope) was used to generate confocal z-stacks of three regions from each cochlea. Image stacks were translated into image-processing software. Each cochlear turn (apex, middle, and base) contained at least 12 IHCs. We chose three visual areas of 20 µm each for each turn of the cochlea, demarcated them with a square, and counted presynaptic ribbons (CtBP2 punctates) of the IHCs that were found around IHCs as well as within nuclei as previously reported (14, 15). We used seven mice from each study group to calculate the average number of presynaptic ribbons per IHC. (page 7, lines 196-205).*

In addition, in the updated manuscript, we recounted synaptic ribbons and presented the number of synaptic ribbons per IHC (page 12, lines 348-356) and changed the values of figure 4 legend (page 25, lines 781, 783,784, 785).

*The CtBP2-positive signal count as determined by IHC revealed that MTX treatment resulted in a significant decrease in the number of synaptic ribbons in all three turns of the cochlea among the group that received only MTX from the cochlea: apex (6±0.9), middle (5±0.7) and base (4±0.7) (Figs 4B, C, D). On the other hand, the AVN-C-treated group maintained high numbers of these synaptic ribbons at the apex (14±0.8), middle (13±1.4), and base (13±0.8) turns (Figs 4B, C, D). The numbers of synaptic ribbons in the FA+MTX-treated (12±1.3, 11±1, and 11±1.1, respectively), AVN-C+FA+MTX-treated group (13±1.4, 12±0.9, and 13±1.3, respectively), and control groups (13±1.1, 11±1.2, and 11±0.9, respectively) at the apex, middle, and base turns and were significant (***p < 0.001) (Figs 4B, C, D) (page 12, lines* 348-356*).*

**Comment:**

**Q1-2. Fig. 2B, C. y-axes should be labeled as “****Threshold (dB SPL)”.**

**Response:**

We are thankful to reviewer for such careful review of our manuscript and for pointing out this issue. We have accordingly updated the fig. 2B, C at y-axes in the revised manuscript.

**Reviewer #2**

**Comment:**

**Q2-1. Abstract – “remarkable” is not the best descriptor for the threshold shift, it would be expected for an ototoxic effect.**

**Response:**

We value the reviewer's critical and helpful feedback on our manuscript. Due to the reviewer's concerns, the term ‘remarkable’ has been removed (page 2, line 39) in the revised manuscript.

*MTX also caused a decrease in wave I amplitude, while AVN-C and FA maintained it at higher levels (page 2, line 39).*

**Comment:**

**Q2-2. Methods – was the total 7 mice in each group or 7 males and 7 females? If the former, what was break-down between males and females. Was this the same in all group? Were different groups of 7 used for each metric (such as detection in body fluids versus hearing tests, scanning EM and hair cell counts, ribbon synapse counting) or was there a total of 7 for all metrics from a condition?**

**Response:**

We are grateful to the reviewer for conducting such a thorough review of our manuscript and for bringing this matter to our concern. We indicated in the present manuscript section of ‘materials and methods’ (page4, line 92) that each group consisted of 7 mice 4 males, and 3 females. We applied this metric for hearing tests, scanning EM, hair cell counts, and ribbon synapse counting.

*Furthermore, inequalities in hearing loss between sexes have significant consequences for knowledge gaps in the translation from non-clinical to clinical settings (17). We used 4 males and 3 females in our study to check for statistical differences between sexes as the difference in sex-based needs for non-clinical versus clinical research can limit a comprehensive knowledge of sex-based mechanistic variables. Some reports showed that women of all ages have superior hearing to men (18). Such disparities may play a role in understanding and explaining clinically significant sex differences, and they are almost certainly essential for developing successful therapeutic treatment options. However, we found no variation in findings attributable to sex differences among the wild type mice studied during our investigations (page 17, line 506-511; page 18, lines 512-514)*.

The detection in body fluids only used 3 as a metric (page 24, line 760) because it was sufficient to get sample for detection.

**Comment:**

**Q2-3 Counting just ribbons and not the combination of ribbon (CTBP2) and receptor (GluR) is not a measure of ribbon synapses. Literature shows there are often ribbons without receptors which would not be a synapse. Ribbon counts are still of value but should be described correctly.**

**Response:**

We appreciate the reviewer's helpful suggestions and direction in improving our manuscript. Hence, in the revised manuscript, we have ensured a high level of clarity regarding the ribbon synapses (pages 7, lines *196-205*). In this paragraph we demonstrated that we focus on presynaptic ribbons as to protect the ribbon synapses would be an effective measure for preventing hearing loss.

*Cochlear turn lengths were determined for each study group. A high-resolution confocal microscope (LSM 800 laser scanning microscope) was used to generate confocal z-stacks of three regions from each cochlea. Image stacks were translated into image-processing software. Each cochlear turn (apex, middle, and base) contained at least 12 IHCs. We chose three visual areas of 20 µm each for each turn of the cochlea, demarcated them with a square, and counted presynaptic ribbons (CtBP2 punctates) of the IHCs that were found around IHCs as well as within nuclei as previously reported (14, 15). We used seven mice from each study group to calculate the average number of presynaptic ribbons per IHC. (page 7, lines 196-205).*

**Comment:**

**Q2-2. The quantitative analysis of outer hair cell loss needs to be better described. How were the regions counted selected to reduce potential for bias? How were counts done? Similarly, how were regions for ribbon counts selected and how were counts done?**

**Response:**

We thank the reviewer for their critical and helpful review of our manuscript. As per the reviewer’s concerns, we have described and incorporated more details about the quantitative analysis of outer hair cell loss (page6, line 171-175) in the revised manuscript.

*The number of outer hair cells in cochleae was quantified. The cochleae were separated into apical, middle, and basal turns, and the hair cells in each turn were counted at a magnification of 500X. For each group, the number of hair cells located in 100 µm cochlear turn length was averaged. If the bundle of stereocilia was missing, a hair cell was considered absent (page6, line 171-175).*

Similarly, the counts of ribbon synapses and regions were done as described earlier (pages 7, lines 196–205)

*Cochlear turn lengths were determined for each study group. A high-resolution confocal microscope (LSM 800 laser scanning microscope) was used to generate confocal z-stacks of three regions from each cochlea. Image stacks were translated into image-processing software. Each cochlear turn (apex, middle, and base) contained at least 12 IHCs. We chose three visual areas of 20 µm each for each turn of the cochlea, demarcated them with a square, and counted presynaptic ribbons (CtBP2 punctates) of the IHCs that were found around IHCs as well as within nuclei as previously reported (14, 15). We used seven mice from each study group to calculate the average number of presynaptic ribbons per IHC. (page 7, lines 196-205).*
