# Supplementary material for: Efficiency of antioxidant Avenanthramide-C on high-dose methotrexate-induced ototoxicity in mice
Source: PLoS One. 2022 Mar 30;17(3):e0266108. doi: 10.1371/journal.pone.0266108 (PMC8967015; doi:10.1371/journal.pone.0266108)
Supplement: Attachment — Submitted filename: CNUNI_5977_Response+to+Reviewers.v18.doc [file pone.0266108.s011.doc]

**Efficiency of Antioxidant Avenanthramide-C on High-Dose Methotrexate-Induced Ototoxicity in Mice**

**PONE-D-21-29604**

Thank you for giving us the opportunity to submit a revised draft of our manuscript titled “Efficiency of Antioxidant Avenanthramide-C on High-Dose Methotrexate-Induced Ototoxicity in Mice” to *PLOS ONE*. We truly appreciate the time and effort of the reviewers for providing their valuable feedbacks on our manuscript. We have been able to incorporate relevant changes to reflect most of the suggestions provided by the reviewers. We have highlighted all the changes in the revised manuscript. Here is a point-by-point response to the reviewers’ comments and concerns.

**Reviewer #2**

**Comment:**

**Q1-1. I have only one question/concern remaining. This is how regions where "squares" for counting ribbons were placed. Was this the same regions in each cochlea assessed. Concern is removing potential for unintentional bias in selection of region to be counted.**

**Response:**

We appreciate the reviewer's detailed feedback for improving our manuscript. Yes, we chose 3 area which were similar distance apart for each cochlear turn (apex, middle and base). We have ensured a high level of clarity in the updated manuscript addressing the counting of synaptic ribbons (page 7, line 202).

We chose three visual areas of 20 µm each for each turn of the cochlea *in the same way for all groups,* demarcated…
